# Health-Related Generative AI Use Alongside and Beyond Professional Care: A Qualitative Content Analysis of Reddit Posts

**DOI:** 10.3390/healthcare14203429

**Published:** 2026-10-14

**Authors:** Joomi Kim, Alex R. Parkhouse, Ricky Leung

**Affiliations:** Division of Social and Behavioral Sciences, School of Public Health, University of Memphis, Memphis, TN 38152, USA

**Keywords:** generative artificial intelligence, large language models, ChatGPT, health information seeking, care-seeking, diagnostic testing, healthcare access, patient-provider communication, Reddit

## Abstract

Background/Objectives: This study examined naturally occurring Reddit posts describing health-related generative artificial intelligence (GenAI) use in relation to professional care. Methods: Of 1822 posts retrieved from two Reddit communities, r/AskDocs and r/ChatGPT (1 November 2024 to 30 October 2025), 291 met the inclusion criteria. Inductive qualitative content analysis generated 47 final codes and five themes. Coding was primarily conducted by one researcher, with team review of boundary cases and independent coding of a random subset. Results: Users described practical, emotional, and relational uses. GenAI use occurred before, in preparation for, and after professional care, and sometimes in lieu of care when care was inaccessible or not pursued. Accounts of diagnostic testing included clinician-informed, clinician-mediated, and independently obtained testing. Users also described concerns about disclosing GenAI use, conflicting information from cross-checking, and barriers to acting on GenAI’s advice to seek care. Conclusions: Clinical and system responses should support discussion of AI-informed concerns and shared decision-making, recognize the clinical work involved in assessing and responding to AI-informed test requests, clarify follow-up pathways after independently obtained test results, and account for barriers to acting on care recommendations.

## 1. Introduction

Generative artificial intelligence (GenAI) is increasingly being used as a source of health information and support, with some users consulting large language models (LLMs) before using conventional search engines [1]. An industry report from OpenAI estimated that approximately 3 in 5 U.S. adults had used AI tools for health-related purposes within the preceding 3 months, including exploring symptoms, understanding medical information, and learning about treatment options [2]. Users have also described turning to GenAI for emotional and mental health support, for concerns ranging from anxiety and depression to relationship difficulties [3].

Health-related GenAI use may extend to decisions about seeking professional care and making sense of information received during care. Cross-sectional surveys have documented users’ reports of changing plans for medical visits and requesting tests or referrals [4,5]. Users have described using GenAI to appraise the severity of symptoms, prepare for consultations, understand physicians’ diagnoses, and explore treatment options [6,7]. From the perspective of uncertainty management theory [8], such uses of GenAI can be understood as ways of managing health-related uncertainty, including whether and how to seek professional care and how to interpret information related to care.

The appropriateness of GenAI’s recommendations matters when users turn to it to guide decisions about professional care. Previous evaluations have raised concerns that GenAI advice may delay needed care by underestimating symptom urgency or contribute to unnecessary healthcare use by overestimating it [9,10]. Similar concerns arise when GenAI suggests diagnostic tests. Some clinical case evaluations have found that LLMs recommend unnecessary diagnostic testing [11,12]. However, these evaluations alone are insufficient to understand how users interpret or act on such recommendations.

Existing research has examined the roles and contexts of GenAI use in relation to professional care through users’ accounts. A Reddit study found that GenAI use was more often described in connection with seeking, continuing, or escalating professional care than with replacing or reducing it [13]. Another Reddit-based study in a mental health context documented use in preparation for therapy sessions and when professional support was unavailable or unaffordable [14]. A longitudinal diary study described patients returning to LLMs to reassess clinicians’ advice and translate recommendations into everyday self-management [15].

The present study contributes to this emerging literature by exploring how users describe health-related GenAI use and its relationship to professional care. The analysis highlights variation in how diagnostic tests are suggested and pursued, including whether and how clinicians are involved.

Reddit provides topic-specific communities, known as subreddits, where users share experiences of illness and healthcare [16] and has been used as a source of naturally occurring data in health communication research [17]. User-initiated posts offer accounts of experiences that users share without prompting from researchers.

Accordingly, this study explores two research questions: (1) How do users describe the use of GenAI tools for personal health concerns? (2) How do users describe GenAI use in relation to decisions about and experiences with professional care?

## 2. Materials and Methods

### 2.1. Study Design

This study employed an inductive, qualitative research design and included a multistep content analysis of user narratives. The codes were derived from the data rather than from a predetermined coding schema. The analysis examined users’ accounts of their experiences; it did not evaluate the clinical accuracy of the AI-generated information they described. This study was reported in accordance with the Standards for Reporting Qualitative Research (SRQR). The completed checklist is provided in Table S1 [18].

### 2.2. Data Ethics

The Institutional Review Board (IRB) at the University of Memphis determined that this project does not involve human subjects as defined by federal regulations (45 CFR 46.102(e)). The IRB further determined that review and approval were not required for this project. As an additional privacy precaution, excerpts were kept brief and some were minimally edited because quoted material from public online posts may be traceable through online searches [19]. These edits were limited to removing non-substantive words or adjusting punctuation, and replacing searchable or highly specific details (e.g., AI tool names or specific medical conditions) with bracketed descriptors, while preserving the original meaning. Usernames, post URLs, and other direct identifiers were also omitted to reduce the risk of reverse identification.

### 2.3. Data Access

Reddit data were obtained through the Reddit for Researchers program, an official academic data access initiative of Reddit. The research team submitted an abstract including a data use plan, which underwent review by the Reddit Research Team. Upon approval, a research data agreement was executed between Reddit and the researchers, and access credentials were granted. Data were accessed through Google BigQuery using credentials provided by Reddit for authorized researchers. The retrieved dataset was exported from BigQuery for screening and subsequent analysis.

### 2.4. Data Collection

Data were retrieved from two public Reddit communities: r/AskDocs, a health-focused community where users seek medical guidance, and r/ChatGPT, an AI-centered community where users discuss their experiences with generative AI tools. Among the 12 candidate subreddits considered across health-focused and AI-focused groups, we selected the subreddit with the highest total post volume within each group, yielding complementary contexts for examining personal health-related GenAI use. The candidate subreddits and their post counts are presented in Table S2.

Posts created from 1 November 2024 to 30 October 2025 were retrieved on 18 May 2026. Keyword filtering was applied to post titles and bodies. Posts were required to contain at least one health-related keyword (health, symptom, doctor, or medical), and at least one GenAI-related keyword (ChatGPT, GPT, Gemini, Claude, or generative AI). Keyword matching was case-insensitive. Posts marked as “[deleted]” or “[removed]” or with null author IDs were excluded by the retrieval query before export; the number excluded was not separately recorded. The unit of retrieval was the original Reddit post; comments were not included because the analysis focused on users’ own experiences rather than the interactions or exchanges that followed the posts. The SQL query used to retrieve posts is provided in File S1. Claude was used to assist with drafting and reviewing the SQL queries used for data retrieval.

### 2.5. Screening and Eligibility

The initial corpus consisted of 285 posts from r/AskDocs and 1537 posts from r/ChatGPT. The research team agreed on the inclusion and exclusion criteria before screening, and the first author screened all posts using these criteria. Cases requiring clarification of the application of the predefined criteria were discussed in team meetings. Nine duplicate posts were removed, leaving 1813 posts for eligibility assessments.

Posts were included when the poster described their own direct use of a GenAI tool for a personal health concern or on behalf of a family member for whom they were providing care. The GenAI use had to be connected to a perceived health risk, symptom, diagnosis, condition, or health-related goal. This criterion distinguished personally relevant health use from general curiosity about AI capabilities.

Posts were excluded when they (1) were keyword-matching false positives with no substantive relevance (e.g., the laboratory abbreviation “SGPT” matching the keyword “GPT”; “healthy” used metaphorically for non-health topics); (2) expressed opinions about AI health use without describing personal experience (e.g., AI ethics discussions); (3) described AI use without personal health motivation (e.g., prompt engineering); (4) described another person’s use of AI rather than the poster’s own direct use; (5) concerned a pet’s health issue; (6) consisted of promotional content; or (7) provided insufficient user-authored detail to characterize the reported GenAI use. When multiple exclusion criteria applied, each post was counted once, under the first reason for exclusion identified during screening. Verbatim AI-generated content within otherwise eligible posts was not coded; only the user-authored narratives were analyzed. Posts without substantive user-authored narrative were excluded.

**Figure 1:**
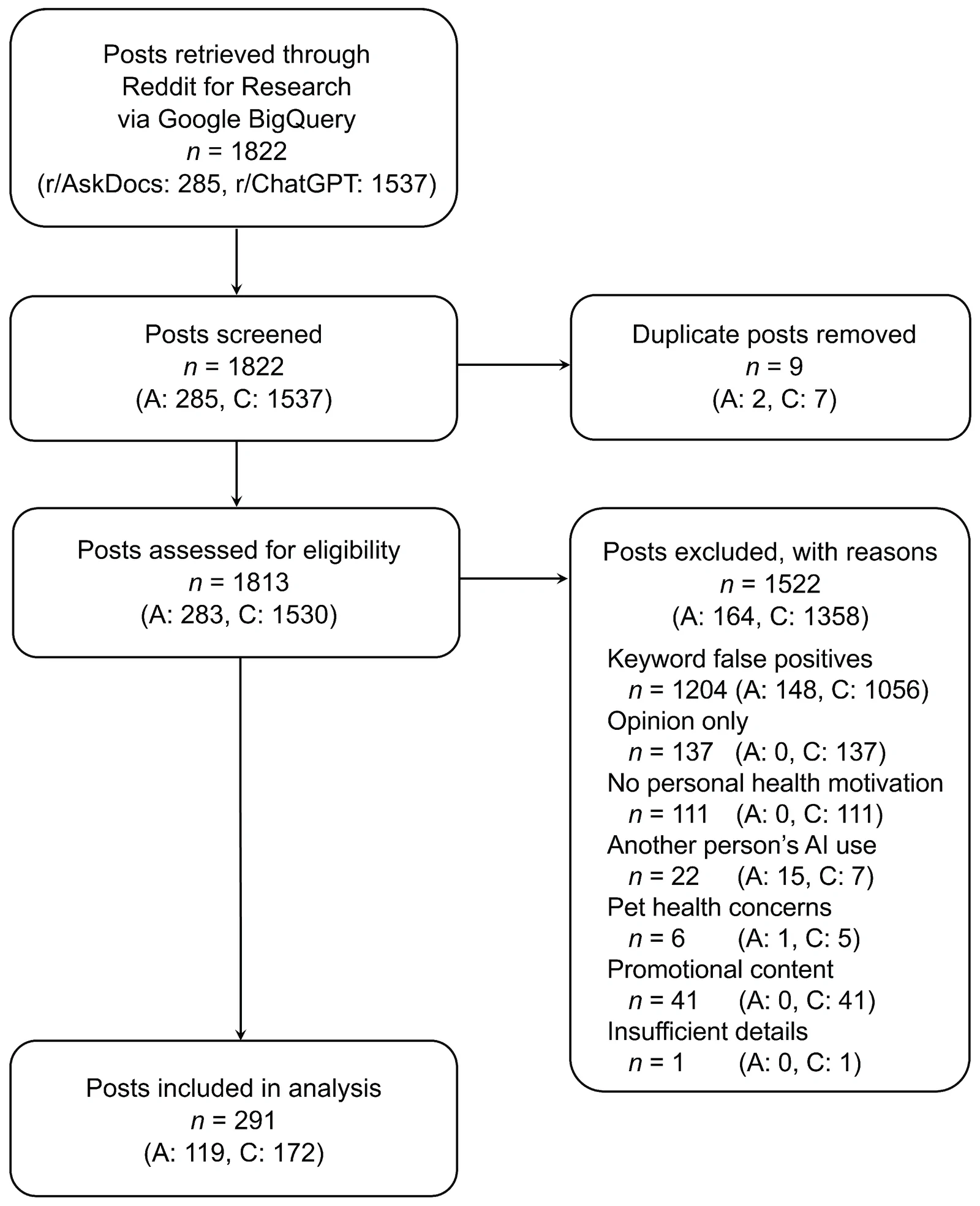
Flow diagram of post-selection. Note: A = r/AskDocs; C = r/ChatGPT.

After screening, 119 posts from r/AskDocs and 172 posts from r/ChatGPT met the inclusion criteria, yielding a final analytical sample of 291 posts. The screening process and reasons for exclusion are shown in Figure 1.

### 2.6. Data Analysis

Data analysis included multiple, systematic steps, informed by the coding procedures described by Corbin and Strauss [20]. First, prior to coding all eligible posts, two members of the research team conducted a pilot coding exercise, drawing on 10 randomly selected posts from each subreddit to align the coding criteria. Posts were numbered separately within each subreddit, and a random number generator was used to select pilot posts from each subreddit.

Next, an open-coding review of all eligible user posts was conducted by the first author. The codes captured what was explicitly stated in each post, including the uses of GenAI, the healthcare circumstances described, and users’ responses to AI-generated information. Motivations, mechanisms, and causal connections were coded only when stated by the poster. A codebook was developed by the first author iteratively and included definitions, inclusion and exclusion criteria, and representative excerpts (Table S3). During the first coding cycle, the first author brought boundary cases to regular team meetings. Uncertain cases were resolved by consensus, and the codebook definitions were revised when necessary. Codes were maintained at a single level. ATLAS.ti Web (version 26, ATLAS.ti Scientific Software Development GmbH, Berlin, Germany) [21] was used by researchers for coding and subsequent analyses. All 291 eligible posts were included in the analysis; repetition in the main codes was observed as coding progressed, but saturation was not used as a stopping criterion or systematically tracked.

In the second, axial coding phase, previously coded material was re-examined to refine the initial codes and explore relationships among categories. For example, excerpts initially coded broadly as AI use were recoded according to the type of use, including interpretation, self-management, and healthcare navigation. The review refined 37 initial codes into 47 final ones. We then examined how these types of use related to the circumstances in which users sought GenAI support, including the timing relative to professional care.

In the final phase, the final codes were organized into broader categories, and themes were developed by examining relationships among codes and recurring patterns relevant to the research questions. For example, codes concerning healthcare access barriers, medical dissatisfaction, and unresolved diagnoses contributed to the theme of unresolved healthcare needs and barriers. These codes described circumstances in which users reported that their healthcare needs had not been met or that their concerns remained unresolved.

After the analysis was completed, intercoder reliability was assessed post hoc. The senior author, who had participated in the pilot coding, independently coded a random sample of 58 posts (20% of the dataset) using the 47-code codebook; the 20 pilot posts were excluded from this sample. The senior author was aware of the overall findings but was blinded to the first author’s post-level codes. Agreement was assessed at the post level, treating the presence or absence of each code in each post as a binary decision. Cohen’s kappa and percentage agreement were calculated by pooling all code-post decisions (1276 decisions for the 22 main codes; 2726 for all 47 codes) using IBM SPSS Statistics for Windows, Version 23.0 (IBM Corp., Armonk, NY, USA) [22]. Agreement was substantial for both the 22 main codes (96.9%; κ = 0.74) and all 47 codes (95.8%; κ = 0.62) [23]. Among the main codes, discrepancies were concentrated in four codes (Legitimacy, Emotional support from AI, AI-suggested diagnosis, and Comparing AIs). Their definitions were clarified to reflect the first author’s existing coding criteria without changing the dataset’s coding (Table S3). Discrepancies in non-main codes largely reflected the senior author’s focus on AI-use-related content rather than the surrounding context. The two coders discussed and resolved all discrepancies through consensus.

Code frequencies represent counts of coded quotations. The numbers of distinct posts and unique authors were also calculated for each final code. These counts were used to describe the distribution of coded content and were not interpreted as measures of prevalence, importance, or association.

Trustworthiness was supported through independent pilot coding and comparison of coding on the pilot posts, regular consensus meetings on boundary cases, iterative codebook development, a post hoc intercoder reliability assessment, and creation and reflexive review of researcher memos throughout. The senior author reviewed the evolving codebook, category boundaries, and candidate themes. The first author kept notes on emerging patterns and relationships among codes throughout the analysis. We also examined negative cases that did not fit the dominant patterns identified in the analysis.

The research team included a researcher with academic training in communication and interaction design and two public health faculty members with doctoral training in sociology, one of whom had substantial experience in conducting qualitative research. The first author’s perspective was informed by prior research on health information overload, confusion, health literacy, and doctor-shopping behavior. This background shaped an interest in how users understand and act on health information and whether GenAI helps or complicates these processes. AI ethics training received through the NIH AIM-AHEAD program also informed attention to the potential risks and limitations of GenAI.

Quotations and descriptions of individual posts are labeled with post identifiers (e.g., C174) to distinguish source accounts. The prefixes “A” and “C” in post identifiers denote r/AskDocs and r/ChatGPT, respectively.

## 3. Results

The 291 included posts were contributed by 286 unique authors. ChatGPT was the most frequently mentioned GenAI tool. Further descriptive characteristics are provided in Table S4.

The research team identified 47 final codes and five themes in the included posts: (1) practical uses of GenAI for understanding and managing health concerns, (2) emotional and relational uses of GenAI, (3) GenAI use in care-seeking, clinician interaction, and diagnostic testing, (4) GenAI use in contexts of unresolved healthcare needs and barriers, and (5) legitimacy concerns, continued use, and cross-checking. Themes 1 and 2 primarily address the forms and purposes of health-related GenAI use (RQ1). Themes 3 and 4 more directly address how GenAI use was situated in relation to professional care (RQ2). Theme 5 cuts across both research questions.

Table 1 presents selected codes relevant to the research questions. Illustrative excerpts are presented in Table 2.

### 3.1. Practical Uses of GenAI for Understanding and Managing Health Concerns

Users asked GenAI to explore possible explanations for symptoms, assess their seriousness, and help them understand health information; the requests sometimes included photographs. For example, one user sent ChatGPT a photograph of painful bumps on their back to get advice (A32).

Beyond one-time information requests, users also used GenAI to track symptoms and health data over time. One user used ChatGPT to track gastrointestinal symptoms in preparation for an upcoming appointment (C998). Some users reported problems with GenAI’s ability to recall previously shared information. One felt that GenAI retained general context but struggled to recall details shared earlier in the conversations (C376), while another asked how to keep personal and health-related information available across conversations (C104).

Users also used GenAI to support day-to-day management of diet, exercise, and other health concerns. One user photographed meals and uploaded the images to GenAI to obtain feedback on their nutritional balance (C291), while another used ChatGPT to help monitor blood glucose (C1246).

### 3.2. Emotional and Relational Uses of GenAI

Users described using GenAI for mental health concerns and also experienced emotional support during GenAI interactions. Mental health-related uses included turning to GenAI for purposes such as venting, coping with stress or distress, or seeking support related to therapy. For example, one user sought support from GenAI between therapy sessions and during nighttime distress (C1386). Emotional support included reassurance, encouragement, validation, and feeling heard, and was not limited to interactions focused on mental health concerns. One user sought reassurance while waiting for a specialist appointment: “my appointment is 1.5 months for now, so I was trying to put my mind at ease” (C563). Reassurance was sometimes temporary. One user reported short-term relief following GenAI use while the underlying uncertainty remained unresolved (A105).

Some users described GenAI in relational terms, calling it a friend or giving it affectionate nicknames. One user who regarded ChatGPT as a close friend reported that conversations were being redirected to GPT-5 and that responses had changed, expressing a sense of loss: “she is not there anymore” (C1405). Other users described intensive use or referred to their use as dependence. One user reported spending more than 12 h a day interacting with AI while paying less attention to sleep, meals, and family interactions (C1535).

### 3.3. GenAI Use in Care-Seeking, Clinician Interaction, and Diagnostic Testing

Users reported using GenAI to identify and evaluate care options and prepare for visits. One user found a facility with an earlier appointment through ChatGPT, instead of waiting six months (A96). Another user asked ChatGPT to help identify additional details about their health concerns that a clinician might need (C935). Some users explicitly linked AI-generated advice to their decisions to seek care or take treatment-related action (C428). In another account, a user expressed reluctance to follow ChatGPT’s recommendation to go to the emergency room, mentioning concerns about complications with workers’ compensation (A285).

AI-generated information also served as a resource for self-advocacy in clinician encounters. One user used AI-informed knowledge to question clinicians and request changes in their care (C1108). These efforts, however, did not always lead to the care they sought. One post reported that several AI systems repeatedly suggested the same diagnosis, while clinicians considered it too rare and declined to test for it despite the user’s continued efforts to obtain further evaluation (C534). At the same time, some users hesitated to disclose their GenAI use, anticipating that clinicians might dismiss information because it came from AI. Some described withholding its source or introducing it indirectly when discussing their concerns.

A notable pattern involved users acting on GenAI suggestions to obtain diagnostic tests, with varying levels of clinician involvement. In some accounts, users brought AI-suggested tests to clinicians for consideration. One user sent their care team an AI-generated message through MyChart listing tests suggested by ChatGPT and Gemini, noting that “they actually ended up ordering the tests” (C950); the test results were later reviewed with a neurologist. In other accounts, users obtained tests with little clinician involvement at the point of testing. One user with persistent symptoms who felt that clinicians had not adequately investigated their concerns mentioned, “some recent tests I did on my own, thanks to suggestion by ChatGPT” (A119). Another obtained a ChatGPT-suggested blood test before a scheduled medical visit (A239).

Users also consulted GenAI to interpret test results. One user uploaded their laboratory results to ChatGPT and asked whether anything had been overlooked (C460). Some users compared AI interpretations with those of clinicians. Across posts, GenAI appeared at different points in diagnostic testing: identifying possible tests, obtaining tests, and interpreting results.

### 3.4. GenAI Use in Contexts of Unresolved Healthcare Needs and Barriers

Some users felt that clinicians had not adequately explained their condition or treatment and turned to GenAI for clarification. One user used ChatGPT to fill “the gap between rushed consultations and my need for understanding” (C1228). Another user left the ER “with zero idea what to do next” and used ChatGPT to understand the tests performed and organize further care-seeking (C1507).

Other accounts described symptoms that remained unexplained despite clinical assessment. One user reported persistent gastrointestinal symptoms despite multiple medical visits and tests that provided no clear answers. The user entered their symptoms into ChatGPT to explore possible causes, then reported researching a condition suggested by ChatGPT, changing their diet, and tracking meals and symptoms (C824).

Users also reported barriers to obtaining professional care, including costs, insurance limitations, geographic barriers, long wait times, or limited mental health or specialty care. One user stated, “I don’t have access to any other form of mental healthcare” (C641). Another compared paying “only $20 a month” for ChatGPT with the costs of therapy and other healthcare services (C1246).

### 3.5. Legitimacy Concerns, Continued Use, and Cross-Checking

Users questioned GenAI’s legitimacy and acknowledged that it could hallucinate and could not diagnose but still found it helpful. One user uploaded MRI images to ChatGPT while awaiting results, acknowledging, “I know AI is not able to diagnose. I probably shouldn’t have done that” (A109). These reservations did not always prevent repeated use. Another user acknowledged that ChatGPT should not be their only source of help but described reporting every meal to it and finding its encouragement helpful (C400).

Users also moved among GenAI, clinicians, online sources, and other AI systems to compare or confirm health information. Some users checked GenAI responses against other sources, whereas others used GenAI to revisit or check information obtained elsewhere. Some r/AskDocs users sought medical opinions from the Reddit community when AI-generated information conflicted with physicians’ advice (A158, A176, A205). Others compared responses across AI systems; for example, one user noted that Gemini suggested a disease as a possible explanation, whereas ChatGPT did not mention it (C764). Another user described using GenAI, searching Google, and seeking professional care, but still reported confusion about possible explanations for their symptoms (A201).

### 3.6. Conceptual Typology

**Figure 2:**
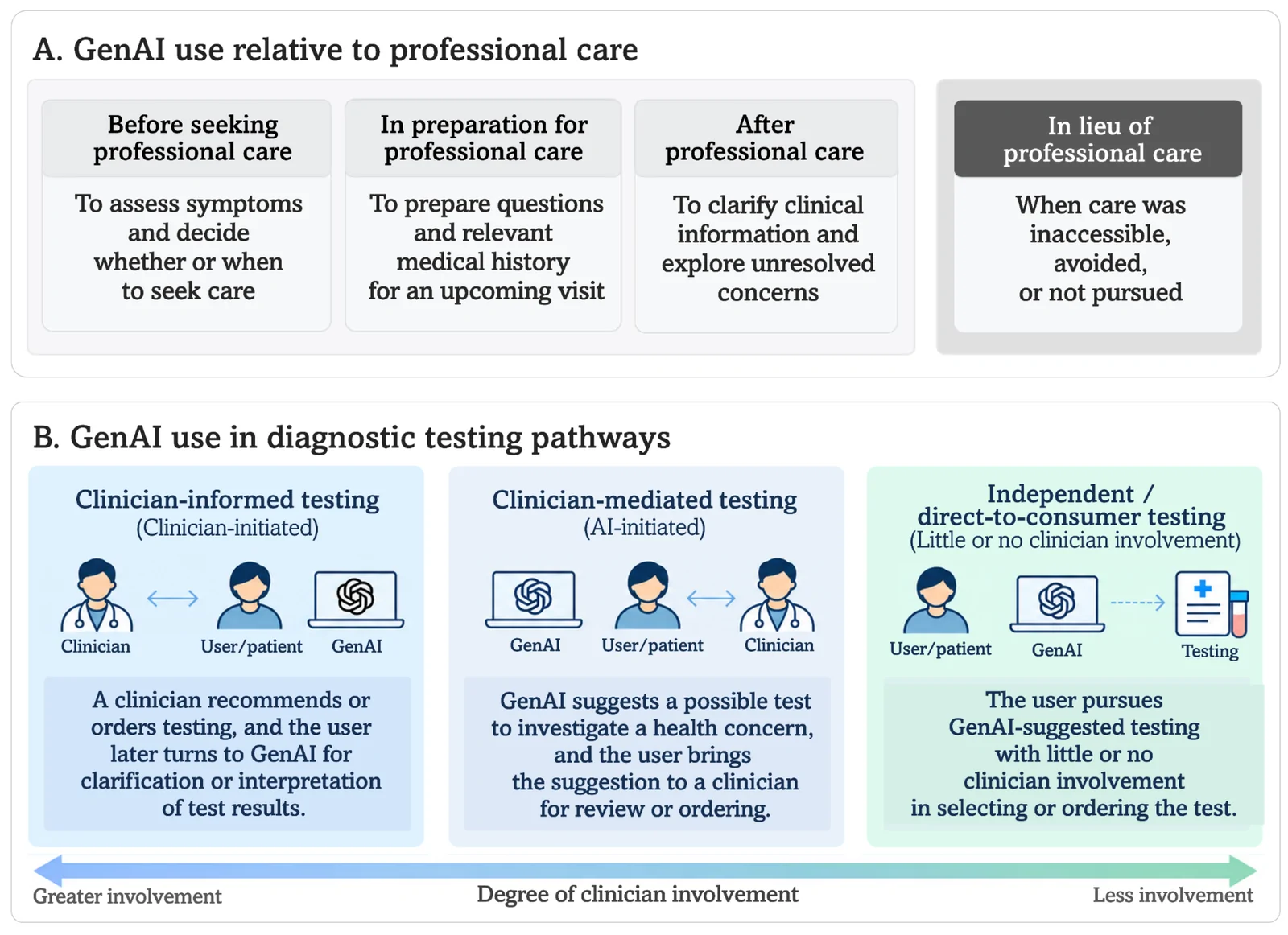
Conceptual typology of GenAI use in relation to professional care and diagnostic testing. Note: The categories are not mutually exclusive, and individual posts may reflect more than one pattern. In Panel (**B**), testing pathways are arranged from greater to less clinician involvement based on how testing was initiated and carried out in the accounts. Bidirectional arrows indicate interactions between users and clinicians, while the dashed arrow indicates users pursuing GenAI-suggested testing with little or no clinician involvement.

We developed a conceptual typology that organizes patterns across the five themes by timing relative to professional care and clinician involvement in diagnostic testing. GenAI use occurred before care, in preparation for care, after clinical encounters, or in lieu of professional care. Testing-related GenAI use included clinician-informed, clinician-mediated, and independent or direct-to-consumer testing. Figure 2 summarizes these patterns and illustrates how GenAI was used both alongside and beyond professional care.

## 4. Discussion

This study examined how users described health-related GenAI use (RQ1) and its relationship with professional care (RQ2) in naturally occurring Reddit posts. RQ1 captured the practical, emotional, and relational roles GenAI served for users, whereas RQ2 highlighted how these uses intersected with care-seeking, clinician interaction, diagnostic testing, and unresolved healthcare needs.

### 4.1. GenAI Use Across Health Management and Emotional Support

Across accounts, users described asking GenAI to interpret symptoms, explain health and treatment information, and support ongoing management of diet, exercise, and symptoms, consistent with earlier research [6,15,24,25]. Tracking health information over time depends in part on how earlier information is carried forward in a conversation. Some users in this study felt that GenAI inaccurately remembered information they had shared. Similar concerns about conversational memory and inconsistent advice were reported in a four-week diary study of patients’ LLM use [15], while simulation-based research found lower performance when task-relevant information was distributed across multiple conversational turns rather than provided in a single complete prompt [26]. Although the present study did not evaluate how accurately GenAI retained or applied earlier health information, users’ expectations that GenAI will remember previous conversations may not align with their reported experiences.

Users described using GenAI to address mental health concerns, as well as feeling heard, understood, and encouraged through their interactions with it, consistent with prior research describing emotional and mental health support through GenAI [14,27,28] and experiences of companionship and therapeutic support [3]. These emotional and relational dimensions are not limited to explicit mental health-related uses. Cao et al. [15], for example, identified emotional support and companionship as part of the broader roles LLMs play across patients’ healthcare-seeking journeys. Some users described GenAI in personal, relational terms. One user in this study expressed a sense of losing a friend after a reported transition to GPT-5, echoing research on experiences of loss following AI model updates and other disruptions to AI companionship [29]. These findings suggest that continuity may be important when GenAI is used repeatedly over time to track personal health information and for ongoing emotional and relational interactions.

### 4.2. GenAI Use in Relation to Professional Care

Users described using GenAI to prepare questions and articulate concerns before clinical encounters, consistent with prior findings on the use of LLMs to support communication with healthcare professionals [6,15]. Bringing AI-informed questions into a consultation was not always straightforward, as some users in this study worried that clinicians might dismiss their concerns because the information came from AI. Fear of negative physician reactions has been identified as a potential barrier to disclosure [5].

Prior research has linked dissatisfaction with care to greater intention to use ChatGPT for mild health concerns [30]. Some users in the present study expressed dissatisfaction with the explanations provided during clinical encounters and turned to GenAI for further clarification. Others used GenAI to explore possible causes of symptoms that remained unexplained after professional care. These accounts reflect different unresolved needs after professional care: insufficient explanation in some cases and continued diagnostic uncertainty in others. This distinction is important because insufficient explanations may be addressed through further clarification, whereas diagnostic uncertainty can persist even after thorough clinical assessments and testing [31]. The present data do not establish whether users distinguish this diagnostic uncertainty from the perceived inadequacy of care when consulting GenAI.

Diagnostic testing showed a clear variation in clinician involvement. Consumer-initiated testing predates the widespread use of GenAI and raised concerns about test selection, result interpretation, and follow-up when professional guidance is limited [32,33]. Users in this study described receiving test suggestions from GenAI and either discussing them with clinicians or obtaining tests independently. Other accounts described using GenAI to interpret the results. Overall, GenAI appeared at multiple points in the testing process, with professional review occurring at different stages or, in some accounts, with little clinician involvement. These differences raise questions about who assesses the need for testing, interprets the results, and coordinates follow-up when users pursue GenAI-suggested tests.

Professional care was difficult to obtain in some accounts because of cost, geographic distance, limited local services, or delays in obtaining care. Users also described turning to GenAI while waiting for appointments or using it to identify an earlier appointment. These accounts suggest that GenAI was used when access was delayed or otherwise difficult, rather than only when professional care was entirely unavailable. This may be consequential because limited healthcare access may reduce opportunities to verify AI responses or correct errors through subsequent consultations [34].

These access barriers may also be relevant to whether users act on GenAI triage recommendations. Some users reported following GenAI’s advice to seek urgent care, while others hesitated because of workers’ compensation concerns. In a study of an AI-based symptom checker, lower income was associated with less safe subsequent care-seeking behavior, even though most recommendations were rated clinically safe [35]. While over- and under-triage concern the clinical appropriateness of recommendations, which this study did not assess, the present accounts highlight a potential gap between receiving GenAI advice to seek care and acting on it.

### 4.3. Continued GenAI Use and Cross-Checking

In this study, users acknowledged GenAI’s limitations while continuing to value its support. Similar combinations of skepticism and perceived usefulness have been reported in previous studies on health information seeking [4,36]. The possibility of acting on inaccurate advice is a concern in this context. In a study by Shekar et al. [37], participants rated low-accuracy AI-generated medical responses similarly to physician-written responses and expressed willingness to follow potentially harmful advice. This raises questions about how users apply their awareness of GenAI’s limitations when evaluating specific recommendations.

Users checked GenAI responses against other sources. Previous studies have similarly described cross-checking GenAI responses against online information, cited sources, and other AI tools [36,38]. The present accounts suggest that cross-checking was not limited to verifying GenAI outputs; users also brought information obtained from clinicians or other sources to GenAI for interpretation or comparison. Blease et al. [38] characterized similar triangulation across AI tools and online sources as potentially occurring within a closed informational loop, rather than through cross-checking against clinicians. In the present accounts, information from clinicians was also included in this cross-checking process.

Cross-checking should not be assumed to resolve uncertainty. In the present study, some comparisons produced conflicting answers, and the posts did not establish whether users ultimately reached more accurate judgments. Consistent with this, Brashers [8] noted that information from different sources can be contradictory and may increase uncertainty. Cross-checking may shift the problem from finding additional information to deciding which source or interpretation to trust. These situations highlight the relevance of AI health literacy, particularly in evaluating AI-generated health information and determining when further verification or professional support is needed [39].

### 4.4. Implications

For clinicians, asking about GenAI use may help identify AI-generated information that contributed to a patient’s understanding, expectations, or preferences for care. The accounts of hesitation about disclosure suggest that clinicians should invite patients to discuss this information without fear of dismissal or judgment. Shared decision-making should make room for patients to explain how AI-generated information has influenced their views and for clinicians to discuss its relevance and limitations in the context of their health concerns. This discussion can inform decisions that consider both clinical evidence and the patient’s goals and preferences.

Healthcare organizations should consider how to incorporate the review of AI-informed test requests into patient portal workflows and staff workload allocation. Assessing whether a requested test is clinically indicated requires weighing its expected diagnostic or management benefit against the risk of low-value testing and downstream diagnostic cascades following false-positive results or incidental findings [33,40]. Therefore, reviewing requests and explaining these decisions may require clinician time even when no tests are ordered. Organizations should designate who reviews incoming requests, define when an appointment is needed rather than a portal reply, and account for the time required to explain testing decisions and communicate follow-up plans.

When users independently obtain testing based on GenAI suggestions, potentially through direct-to-consumer (DTC) services, there may be no designated clinician to assess the results in the context of their symptoms and medical history and coordinate any necessary follow-up. DTC testing providers should clarify who is responsible for reviewing results and provide a clear route to clinical care when further evaluation is needed, particularly for users without an established healthcare provider.

Public health education should support AI health literacy through practical examples of how to evaluate GenAI advice, assess conflicting information, and decide when professional review is needed. These examples could focus on decisions regarding seeking care, obtaining diagnostic testing, or interpreting test results. Education should also explain the limitations of GenAI and precautions relevant to specific health uses. For ongoing health management, this could include encouraging users to check whether responses accurately reflect the health information they shared.

Such guidance should also be feasible for users with limited access to professional care. Recommendations to “consult a clinician” may be difficult to follow when users face limited local services in rural areas, lack of insurance, unaffordable care costs, or long waits for mental health services. These barriers raise an equity concern: users who turn to GenAI in place of professional care they cannot access may also have fewer opportunities to obtain clinical assessment of their concerns. Policy responses should address financial and service availability barriers so that users can obtain clinical assessment and follow-up when needed, including professional review of concerns arising from GenAI use.

### 4.5. Strengths, Limitations, and Future Research

This study has several strengths. First, it examines naturally occurring user accounts, allowing the analysis to capture the experiences and concerns expressed by users in their own terms. Second, it connects GenAI use with specific health activities, including preparation for consultations, seeking explanations after professional care, and diagnostic testing. The testing-related accounts further captured variation in professional involvement, from discussing AI-suggested tests with clinicians to obtaining tests independently and using GenAI to interpret results. Third, this study includes two Reddit communities with different focuses. This allowed GenAI-related health experiences to be examined in both a health-focused and an AI-focused community.

This work is not without limitations. The data were limited to users who publicly shared their health-related GenAI experiences. Experiences that prompted users to seek advice or share their reactions may have been more prominent in the data. Posts deleted or removed, or with null author IDs, were excluded at retrieval, potentially introducing survivorship bias. Sensitive health experiences and accounts removed under subreddit rules may therefore be underrepresented. The posting norms of the two selected communities may also have shaped which experiences users discussed and what they emphasized. These selection and contextual factors limit the transferability of the findings to other online communities and GenAI users more broadly. The keyword-based retrieval strategy may have underrepresented relevant posts omitting the selected health terms in r/AskDocs or GenAI-related terms in r/ChatGPT, potentially influencing the distribution of GenAI uses and care-related experiences captured in the analysis.

Posts may provide incomplete or selective descriptions of users’ experiences. The analysis focused on explicitly stated content and may have missed implicit motivations, meanings, or decision-making processes. Comment threads, peer feedback, and subsequent posts were excluded, limiting insight into how users’ understanding or actions developed after the original post. The accuracy of these accounts, the clinical circumstances described, and subsequent outcomes could not be independently verified. The research team did not include clinicians; clinician-, testing-, and diagnosis-related codes were not reviewed for clinical appropriateness, which may have limited interpretation of clinical nuances in users’ accounts. Reported connections between GenAI use and care-related actions cannot establish causality. Users’ demographic characteristics and healthcare contexts were also not systematically established, limiting comparisons across population groups and healthcare systems.

This study’s broad exploratory scope limited in-depth examination of experiences within specific health conditions or clinical contexts. Although borderline cases at the screening stage were resolved through consensus discussion, screening was not conducted independently by two reviewers. The dataset was coded by a single researcher, and intercoder reliability was assessed post hoc in a 20% subsample. The second coder had participated in the pilot coding and was aware of the overall findings, which may have inflated agreement. Disagreements were concentrated in four codes. Their definitions were clarified to reflect the criteria applied in the analysis, but agreement on these codes was not reassessed after clarification. Code frequencies represent coded quotations and should not be interpreted as prevalence estimates. Finally, the findings may not apply to later models or health-specific products. GenAI technologies also changed during the study period, which may have shaped users’ experiences and expectations over time. The findings therefore reflect a rapidly evolving technological context rather than a single, stable generation of GenAI systems.

Accordingly, future research should follow health-related GenAI use over time to examine subsequent decisions and outcomes, including among users facing barriers to professional care. Building on the testing-related accounts in this study, further work should examine how users move from GenAI suggestions to obtaining tests and interpreting results, and when professional reviews occur. Observational studies complemented by follow-up interviews could examine how users evaluate GenAI responses and whether cross-checking changes their understanding or decisions. Future work could also examine comment threads in r/AskDocs, particularly replies from verified clinicians, to explore how clinicians respond to users’ AI-informed questions and requests to reconcile GenAI responses with physicians’ advice.

## 5. Conclusions

GenAI was used to prepare for professional care, understand concerns that remained unresolved after care, and obtain support when professional care was difficult to access. Testing-related accounts showed variation in clinician involvement, from discussing AI-suggested tests with clinicians to independently ordering and obtaining tests. Clinicians should encourage patients to discuss their GenAI use and incorporate their questions and preferences into shared decision-making. Healthcare organizations should establish procedures for reviewing requests for AI-suggested tests, and DTC testing providers should clarify responsibility for result review and follow-up. Users facing barriers to professional care should also be offered accessible pathways to clinical evaluation.

## Figures and Tables

**Table 1:** Selected final codes and frequencies of coded quotations by subreddit.

Final Code	Coded Quotations		
	r/Ask Docs	r/Chat GPT	Total *n*
AI use for interpretation	31	31	62
AI use for mental health	3	46	49
AI use for symptom appraisal	30	17	47
AI use for recommendation	12	26	38
AI use for self-management	3	30	33
AI use for verification	12	6	18
AI use for tracking health data	3	12	15
AI use for medical visit preparation	2	13	15
AI use for healthcare navigation	2	5	7
AI use linked to healthcare seeking (explicitly stated)	6	18	24
AI use linked to treatment actions (explicitly stated)	4	6	10
AI-suggested diagnosis	23	24	47
Self-diagnosis	10	2	12
Unresolved diagnosis	8	7	15
Healthcare access barrier	13	19	32
Medical dissatisfaction	11	15	26
Legitimacy	24	52	76
AI-physician disagreement	9	5	14
Comparing AIs	1	13	14
Emotional support from AI	2	17	19
Personification of AI	2	15	17
AI overreliance	0	18	18

Note. Frequency represents the number of coded quotations. Selected codes are presented based on their relevance to the research questions and contribution to theme development. The counts of coded quotations, posts, and unique authors for all final codes are provided in Table S5.

**Table 2:** Overarching themes and illustrative accounts.

Themes	Illustrative Accounts
Practical uses of GenAI for understanding and managing health concerns	“I explained my symptoms to ChatGPT and it recommended I immediately go to the hospital” (C174) “I am currently using [AI] to try and track my symptoms for [a health issue] and upcoming appointment” (C998) “I take photos of what I’m eating and [AI] recognizes how balanced the meal is” (C291)
Emotional and relational uses of GenAI	“What ChatGPT has helped me is giving me encouragement to become healthy” (C942) “It calms me, validates me, helps me understand my own fears” (C688) “ChatGPT has become a friend to me” (C1386)
GenAI use in care-seeking, clinician interaction, and diagnostic testing	“[AI] insists on [a disease] and that I need to rush back to hospital, which I did” (C428) “[AI] helped me advocate for myself in language that made the doctors listen” (C623) “Before visiting doctor, I went to a lab for blood tests [AI] told me to do” (A239)
GenAI use in contexts of unresolved healthcare needs and barriers	“I was recently out of the ER … with zero idea what to do next. [AI] is helping me to organize, and to explain what was done” (C1507) “Multiple clinicians … and no one could figure it out. Today I ran it past [AI] and it said it was [a diagnosis]. Suddenly everything makes sense” (C941) “I asked [AI] about it earlier … But I’m still unsure. I can’t see a doctor right now—I live in a remote rural area with limited access” (A148)
Legitimacy concerns, continued use, and cross-checking	“I know AI is not able to diagnose. I probably shouldn’t have done that” (A109) “I know [AI] shouldn’t be my only resource of help, but is a 24/7 support… I tell [AI] about every meal I eat… [AI] is helping me a lot” (C400) “GPT never even mentioned [a disease] once, whereas [another AI] figured it out in a single prompt” (C764)

Note. Excerpts illustrate selected aspects of each theme. Bracketed substitutions were used where necessary to reduce search-based traceability while preserving meaning.

## Data Availability

Restrictions apply to the availability of these data. The Reddit data analyzed in this study were accessed through the Reddit for Researchers program under a research data agreement with Reddit and cannot be shared publicly or redistributed by the authors. Researchers seeking access to the source data must apply directly through the Reddit for Researchers program. The SQL queries used to retrieve the data are provided in Supplementary File S1.

## Supplementary Materials

The following supporting information can be downloaded at https://www.mdpi.com/article/10.3390/healthcare14203429/s1, Table S1: Standards for Reporting Qualitative Research (SRQR) checklist; Table S2: candidate subreddits considered for selection and their post counts; Table S3: codebook with definitions and illustrative excerpts; Table S4: descriptive characteristics of the included Reddit posts (n = 291); Table S5: number of coded quotations, posts, and unique authors for each final code; File S1: SQL queries used to retrieve posts meeting the study criteria.
