# Supplementary material for: Health-Related Generative AI Use Alongside and Beyond Professional Care: A Qualitative Content Analysis of Reddit Posts
Source: Healthcare (Basel). 2026 Oct 14;14(20):3429. doi: 10.3390/healthcare14203429 (PMC13704374; doi:10.3390/healthcare14203429)
Supplement: Supplementary file 1 [file healthcare-14-03429-s001.zip › healthcare-4611993-supplementary.pdf]

Supplementary Materials

Table S1. Standards for Reporting Qualitative Research (SRQR) checklist

Topic	Reporting item	Page No.
Title	Concise description of the nature and topic of the study identifying the study as qualitative or indicating the approach (e.g., ethnography, grounded theory) or data collection methods (e.g., interview, focus group) is recommended	1
Abstract	Summary of key elements of the study using the abstract format of the intended publication; typically includes background, purpose, methods, results, and conclusions	1
Problem and rationale	Description and significance of the problem/phenomenon studied; review of relevant theory and empirical work; problem statement	1–2
Purpose / research questions	Purpose of the study and/or specific objectives or questions	2
Qualitative approach	Qualitative approach (e.g., ethnography, grounded theory, case study, phenomenology, narrative research) and guiding theory if appropriate; identifying the research paradigm (e.g., postpositivist, constructivist/ interpretivist) is also recommended	2
Researcher role/ reflexivity	Researchers' characteristics that may influence the research, including personal attributes, qualifications/experience, relationship with participants, assumptions, and/or presuppositions; potential or actual interaction between researchers' characteristics and the research questions, approach, methods, results, and/or transferability	6
Study context	Setting/site and salient contextual factors	3–4
Sampling strategy	How and why research participants, documents, or events were selected; criteria for deciding when no further sampling was necessary (e.g., sampling saturation)	3–4
Ethics and human-subjects considerations	Documentation of approval by an appropriate ethics review board and participant consent, or explanation for lack thereof; other confidentiality and data security issues	2-3
Data collection procedures	Types of data collected; details of data collection procedures including (as appropriate) start and stop dates of data collection and analysis, iterative process, triangulation of sources/methods, and modification of procedures in response to evolving study findings	3–4
Collection tools / technology	Description of instruments (e.g., interview guides, questionnaires) and devices (e.g., audio recorders) used for data collection; if/how the instrument(s) changed over the course of the study	2–3
Units of study	Number and relevant characteristics of participants, documents, or events included in the study; level of participation (could be reported in results)	4
Data processing	Methods for processing data prior to and during analysis, including transcription, data entry, data management and security, verification of data integrity, data coding, and anonymization/de-identification of excerpts	3–4
Data analysis	Process by which inferences, themes, etc., were identified and developed, including the researchers involved in data analysis; usually references a specific paradigm or approach	4–5

Techniques to enhance trustworthiness	Techniques to enhance trustworthiness and credibility of data analysis (e.g., member checking, audit trail, triangulation)	5-6
Synthesis and interpretation	Main findings (e.g., interpretations, inferences, and themes); might include development of a theory or model, or integration with prior research or theory	6–10
Links to empirical data	Evidence (e.g., quotes, field notes, text excerpts, photographs) to substantiate analytic findings	6–10
Connection to prior work and implications	Short summary of main findings; explanation of how findings and conclusions connect to, support, elaborate on, or challenge conclusions of earlier scholarship; discussion of scope of application/generalizability; identification of unique contribution(s) to scholarship in a discipline or field	10–13
Limitations	Trustworthiness and limitations of findings	13–14
Conflicts of interest	Potential sources of influence or perceived influence on study conduct and conclusions; how these were managed	15
Funding / support	Sources of funding and other support; role of funders in data collection, interpretation, and reporting	15

Table S2. Candidate subreddits considered for selection and their post counts

Community focus	Subreddit	Number of posts[1]	Selected
Health	r/AskDocs	69,295	Yes
Health	r/medical_advice	31,549	No
Health	r/DiagnoseMe	12,329	No
Health	r/medical	8,948	No
Health	r/Health	3,218	No
Health	r/medicine	1,765	No
GenAI	r/ChatGPT	83,347	Yes
GenAI	r/ClaudeAI	17,892	No
GenAI	r/OpenAI	13,615	No
GenAI	r/singularity	8,463	No
GenAI	r/GeminiAI	7,978	No
GenAI	r/ArtificialInteligence	7,284	No

Note: [1] Post counts include all posts available in Reddit for Researchers for November 1, 2024, through October 30, 2025, as retrieved on September 29, 2026, without application of keyword or eligibility filters.

Table S3. Codebook with definitions and illustrative excerpts

Final code	Definition	Inclusion criteria	Exclusion criteria	Illustrative excerpt
AI dissatisfaction	The user expresses a negative evaluation of AI's performance, accuracy, or depth, distinct from a specific factual error.	Explicit negative evaluation of the AI interaction itself.	A specific factual error or fabricated data (code as AI limitation instead); general hedging or self-disclaimer about AI's trustworthiness (code as Legitimacy instead).	"now [upgraded version of the AI] sucks" (C1241)
AI-suggested diagnosis	The user describes AI as explicitly suggesting or proposing a specific named condition or diagnosis.	A specific named condition or diagnosis is explicitly attributed to AI, including statements such as "AI diagnosed me with [condition]".	AI evaluates symptoms without suggesting or identifying a specific named condition; code as AI use for symptom appraisal where applicable.	"I get a diagnosis by [AI] of [a disease]" (C428)
AI use linked to healthcare seeking (explicitly stated)	AI's output is explicitly described as having influenced the user's decision to seek or avoid medical care.	A clear textual link between AI output and a care-seeking decision or action, such as seeking, returning to, delaying, or avoiding professional care	AI and a medical visit are both mentioned but no causal link is stated between them.	"[AI] insists on [a disease] and that I need to rush back to hospital, which I did" (C428)
AI use linked to treatment actions (explicitly stated)	AI's output is explicitly described as having influenced a change in medication, treatment, or self-care regimen.	The user describes switching medications, dosages, or treatment approaches as a result of AI's suggestion.	—	"It said [a medicine] wouldn't help and to take [another medicine] so I've begun taking that before bed" (A175)
AI limitation	AI is described as producing an actual factual error, fabrication, inconsistency, or unsafe output.	A concrete instance of AI's output being wrong, fabricated, inconsistent, or unsafe is described.	The user's general distrust without a specific described error (code as Legitimacy instead).	"I've tried with 4o, 4.1, and 5, and seemingly it ignores the fact that I have serious and specific health complications and actively gives advice that could further endanger my kidneys" (C1407)

AI overreliance	The user explicitly describes dependence on, excessive use of, or unusually high reliance on AI.	Explicit statements of excessive use, high reliance, or dependence.	—	"I began spending more than twelve hours a day talking to the machine." (C1535)
AI use for healthcare navigation	AI is described as providing information that helps the user identify relevant specialists, care pathways, or options the user was not previously aware of.	AI names a type of specialist or provider, or care pathway, the user had not considered, or helps locate faster or alternative access points.	—	"I actually used [AI] in the interim to find a place that can get me in much sooner than 6 month" (A96)
AI use for interpretation	The user provides existing health-related data or documents (test results, imaging, medical records, or insurance/coverage documents) to AI for explanation or interpretation.	Existing or prior medical data is described as having been input to AI for interpretation.	AI used to track new, ongoing data over time (code as AI use for tracking health data instead).	"I uploaded all my lab results, … to [AI]. Told it my current issues of [symptoms] and asked it to look over all the information" (C460)
AI use for medical visit preparation	AI is used prior to a medical appointment to organize questions, summarize history, or otherwise prepare for the visit.	Explicit description of pre-visit preparation activity using AI.	AI use occurring only during the encounter without a pre-visit preparation component	"It offered me a summary to take to the doctor and a list of questions to be sure to ask, … and OMG. This is the best thing EVER." (C396)
AI use for mental health	AI is used for a mental health-related purpose or to address emotional or psychological distress.	Mental health is the explicit purpose or subject of the AI interaction.	—	"I mostly use [AI] for emotional support - comforting, venting, coping." (C1377)
AI use for recommendation	The user seeks or receives AI-generated advice or recommendations about what action to take for a health concern.	Seeking or receiving advice about what action to take for a health concern, such as whether to seek care, pursue diagnostic testing, or use a treatment or medication. The user does not need to	—	"ChatGPT recommended that I try applying [a medicine] on my lips." (A235)

		have acted on the recommendation.		
AI use for self-management	The user describes using AI to manage a health condition, symptoms, or health-related goals through ongoing self-care or day-to-day management.	Use of AI to support symptom management, diet, sleep, exercise, routines, coping strategies, medication or supplement management, or other ongoing self-care activities.	Seeking or receiving a recommendation without describing its use in ongoing self-care or day-to-day management; diagnostic or explanatory uses without a management component.	"I take a photo of what I'm eating and [AI] automatically recognizes what I'm eating, how many calories, the food contents, …. I have to go and makes suggestions" (C291)
AI use for symptom appraisal	AI is used to evaluate or make sense of symptoms, including their possible causes, significance, severity, or urgency	Symptoms are described as having been presented to AI, and the AI response evaluates or interprets their possible causes, significance, severity, or urgency	A named diagnosis suggested by AI without an explicit description of symptom appraisal.	"I explained my symptoms to [AI] and it recommended…" (C174)
AI use for tracking health data	AI is used as the medium for ongoing or longitudinal logging of health-related data over time.	Repeated or regular logging behavior using AI as the recording tool is explicitly described.	One-time interpretation of already-existing data (code as AI use for interpretation instead); tracking performed independently without AI as the logging medium.	"I am currently using [AI] to try and track my symptoms for [a health issue] and upcoming appointment." (C998)
AI use for verification	AI is used specifically to confirm or validate health information, including medication interactions, prior information, or a healthcare professional's assessment or decision	Explicit use of AI to cross-check medication interactions, health information, or clinical advice, assessment, or decisions.	New diagnoses or explanations generated by AI; interpretation of test results or medical records; general health information seeking; advice or recommendations about what to do.	"I even sent in videos of his breathing just to make sure things looked okay. Every answer it gave lined up with what our doctors confirmed." (C604)
AI use while waiting for appointment	AI is used during the interval while the user is waiting for an upcoming medical appointment.	AI use is explicitly framed as occurring during a waiting period before a scheduled appointment.	—	"my appointment is 1.5 months for now, so I was trying to put my mind at ease. But ChatGPT offered very few viable options" (C563)

AI-physician agreement	AI-suggested diagnosis or AI's interpretation explicitly aligns with a physician's stated diagnosis or opinion.	Both an AI-derived conclusion and a physician's matching conclusion are stated, with the alignment made explicit.	—	"It told me I may have [a disease] … I showed her the results from the hospital and she agreed." (C716)
AI-physician disagreement	AI-suggested diagnosis or AI's interpretation explicitly conflicts with a physician's stated diagnosis or opinion.	Both an AI-derived conclusion and a physician's differing conclusion are stated, with the discrepancy made explicit.	Disagreement between patient and physician without AI involvement (code as Patient-physician conflict instead).	"all doctors say the diagnosis AI endorses is too rare, not relevant and refuse to test for it." (C534)
Clinician diagnosis	A specific diagnosis given by a physician or clinician is named.	A named diagnosis attributed to a clinician is explicitly stated.	Self-derived or AI-derived diagnostic possibilities not confirmed by a clinician (code as Self-diagnosis or AI-suggested diagnosis instead).	"I was finally diagnosed with [a disease] after imaging, physical therapy, two years of chiropractic care" (A61)
Comparing AI with Google	The user explicitly compares AI's output with information obtained from a Google search for the same health query.	Both AI and Google are named as sources for the same query, with their outputs compared or contrasted.	—	"[AI] combines everything I write and understands what I want, google is chaos"(C1099)
Comparing AIs	The user explicitly compares the output of two or more AI tools for the same health query.	Two or more named AI tools are compared on the same query with their results contrasted.	Comparisons with a Google search are coded as Comparing AI with Google.	"GPT never even mentioned [a disease] once, whereas [another AI] figured it out in a single prompt" (C764)
Confusion	The user expresses a cognitive or epistemic state of being unable to understand or reconcile conflicting information.	Explicit expression of confusion or inability to make sense of the situation.	Do not code under Emotional response, as confusion is a cognitive/epistemic state rather than an emotion.	"I tick a lot of the boxes for [a disease] or [another disease]. They could all be the same thing-I don't even know anymore, I'm so confused." (A201)
Diagnostics	Tests or diagnostic tools/procedures used (e.g., MRI,	A specific test or procedure is named as having been performed.	—	"She sent me for a liver scan and some blood test" (C716)

	blood work, biopsy, EMG, DEXA, CT, endoscopy).			
Doctor shopping	The user or the family member seeks out or switches to additional or different physicians.	A concrete action of consulting or switching to another physician or specialist is described.	Dissatisfaction is expressed but no actual switching or seeking of another provider is described (code as Medical dissatisfaction only).	"I didn't really trust his diagnose, so I went to another one in December that said nothing is wrong with me" (A124)
Emotional response	The user directly expresses their own emotional or affective state using explicit emotion-denoting language.	An explicit emotion word or phrase describing the user's own felt state is present in the text.	Cognitive states such as confusion (code as Confusion instead); situational or background stressors not framed as a felt emotion (consider Psychological vulnerability instead).	"I'm at lost and desperate." (A79)
Emotional support from AI	AI is described as having provided comfort, validation, or emotional reassurance to the user.	AI's act of comforting, validating, or reassuring is explicitly described.	—	"It calms me, validates me, helps me understand my own fears" (C688)
Health behavior	Lifestyle or behavioral changes made by the user, whether or not directly tied to a specific AI recommendation.	A concrete behavior change is described.	—	"I began avoiding fried foods, eliminated sugar completely." (C18)
Healthcare access barrier	Structural barriers to accessing professional healthcare are described - cost, insurance, wait times, geography, or time constraints.	A specific structural barrier is explicitly named as limiting access to care.	Personal or psychological reluctance to seek care not tied to a structural barrier (code as Healthcare avoidance instead).	"I asked [AI] about it earlier… But I'm still unsure. I can't see a doctor right now - live in a remote rural area with limited access" (A148)
Healthcare avoidance	The user explicitly describes avoiding or wanting to avoid professional care for personal or psychological reasons rather than structural or access barriers	Avoidance or desire to avoid professional care is explicitly attributed to a psychological or personal reason rather than cost, distance, or wait time.	Avoidance attributed to a structural barrier (code as Healthcare access barrier instead).	"I have [a disease] and have a lot of strep throat test trauma so I have a bad gag reflex so the ENT is terrifying to me that I really want to avoid." (A175)

Legitimacy	The user explicitly questions, qualifies, or reflects on the credibility, appropriateness, or limitations of using AI for health-related purposes or relying on AI-generated health information	Self-disclaimer language, expressions of uncertainty or skepticism about AI's accuracy; posts where AI output is explicitly presented as something to be verified or questioned; (e.g. "ChatGPT said XXX - is this accurate?")	Do not code Legitimacy solely because a factual error occurred; code AI limitation unless the user also explicitly reflects on AI's broader uncertainty or limitations	"I know AI can't replace a therapist." (C25)
Limited social support	The user describes a lack of people in their life to confide in or rely on, distinct from a structural healthcare access barrier.	Explicit description of social isolation or lack of confidants, used as context for AI use.	Do not code as Healthcare access barrier, which is reserved for structural barriers to professional care.	"Because no one else would be here for me" (C1277)
Medical dissatisfaction	The user expresses a negative evaluation of a physician's or the healthcare system's diagnosis, treatment, attitude, or responsiveness.	A negative judgment about what a doctor or system did or failed to do is explicitly stated.	—	"NOBODY in my medical team has been willing to offer me any suggestions other than to take pain meds" (A100)
Medical treatment	A specific medication, procedure, or intervention received is named.	A named medication, dosage, or procedure is described as having been administered or prescribed.	—	"I had to stay in the hospital for a week getting IVs constantly and being monitored" (C174)
Medical visit	A visit to a doctor, specialist, ER, or other healthcare provider/setting is described.	A specific visit or encounter with a healthcare provider is explicitly mentioned.	Do not code the absence of a visit unless explicitly stated by the user (see No medical visit).	"I've been going to psychologists for many years now. I've had [number] psychologists and [number] psychiatrists overall" (C306)
Mental health symptoms	The user explicitly states psychological or psychiatric conditions	A specific mental health condition (e.g. OCD, panic attack) is explicitly named.	Do not include short-term feelings caused by a specific situation; code them as Emotional response	"[A mental health condition] has really taken a toll on me for a while…. I've been in a living hell for a long time" (C382)

No medical visit	The user explicitly states that they did not seek or attend, or have not sought or attended, a medical visit.	An explicit statement that no visit occurred or was sought is present in the text.	Do not infer absence of a visit simply because none is mentioned.	"I've never been to a therapist" (C341)
Outcome	An explicit, stated result of AI use or of the broader health situation is described, whether positive or negative.	A concrete stated result or change in health status or situation following AI use or care-seeking.	—	"From [specific date] to [specific date], I lost around [number] kg…and significantly lowered my blood sugar levels." (C18)
Patient-physician conflict	Conflict or disagreement between the patient and a physician, not necessarily involving AI directly.	Explicit description of disagreement, dismissal, or conflict between patient and physician.	When the conflict is specifically about AI's output diverging from the physician's, code as AI-physician disagreement.	"she is INSULTED that I would insinuate that she didn't know enough about [a disease]" (A90)
Paying for AI	The user explicitly references paying for an AI subscription or service in the context of health use.	Cost of AI access is explicitly mentioned, often in comparison to the cost of professional care.	—	"By paying only $20 a month I've got [AI] working with me and my medical records on my [a diagnosis]" (C1246)
Perceived AI benefit	The user expresses a positive evaluation of or satisfaction with AI itself	A positive evaluation of AI's helpfulness or value is explicitly stated by the user	A concrete, measurable change in health status or situation is the focus (code as Outcome instead). When a positive evaluation of AI concerns comfort, validation, or reassurance, it is coded as Emotional support from AI.	"That's when I realized [AI] could be an incredibly useful tool" (C404)
Personification of AI	The user describes or relates to GenAI as occupying a human-like social, relational, or professional role. Examples include describing AI as a doctor, therapist, friend, or companion.	Explicitly describes or refers to AI as occupying a human-like social, relational, or professional role (e.g., therapist, friend)	—	"[AI] has become a friend to me" (C1386)
Physical symptoms	Self-reported physical symptoms explicitly stated by the user.	A specific symptom is explicitly named or described by the user as	Do not code symptoms that are only implied or that appear solely in an AI-generated response without the	"I've had low back pain for over a decade" (C422)

		something they are experiencing.	user confirming they experienced them.	
Privacy concern	The user expresses concern about data storage, breach, or exposure of personal health information shared with AI.	Explicit concern about data security or privacy related to AI use is stated.	—	"I'm just genuinely worried that there might be a data breach … and all my inputs could become public"(C25)
Psychological vulnerability	A situational or background psychological or contextual vulnerability is described, distinct from a formally named symptom.	A vulnerability factor or contextual stressor is described as background or context, not as a felt emotion in the moment.	Felt emotional states described as a direct reaction (code as Emotional response instead).	"I grew up in borderline poverty in [a country] …lived through trauma and assault."(C1535)
Self-diagnosis	The user explicitly states a self-derived possible diagnosis or describes self-research leading to a diagnostic conclusion.	The user explicitly identifies a possible diagnosis based on their own interpretation or non-AI self-research.	When AI is the source of the diagnostic suggestion (code as AI-suggested diagnosis instead).	"I personally think it could be something like [a diagnosis], or [another diagnosis]" (A204)
Self-medication	The user manages symptoms through over-the-counter medication or home remedies, often alongside AI consultation.	Specific OTC medication or home remedy use for symptom management is described.	—	"I tried a few home remedies" (A148)
Unresolved diagnosis	The user describes multiple or ongoing medical evaluations without reaching a clear diagnosis.	Explicit statements that testing or evaluation has been inconclusive over an extended period.	Use Medical dissatisfaction instead when the issue is physician attitude or treatment quality rather than the absence of diagnostic clarity itself (both may co-occur).	"But they all ended up shrugging and saying they don't know the cause" (C1518)

Note: Bracketed substitutions were used where necessary to reduce search-based traceability while preserving meaning. Multiple codes could be applied to the same passage when the criteria for each code were met.

Table S4. Descriptive characteristics of the included Reddit posts (n = 291)

Characteristic	n (%)
**Subreddit**	
r/AskDocs	119 (40.9)
r/ChatGPT	172 (59.1)
**Authors**	
Unique authors	286 (100.0)
Repeat authors	5 (1.7)
**GenAI tool mentioned**	
ChatGPT/GPT only	276 (94.8)
Gemini only	5 (1.7)
Claude only	1 (0.3)
Multiple tools	8 (2.7)
Unspecified GenAI tool	1 (0.3)
**Posting month**	
November 2024	15 (5.2)
December 2024	9 (3.1)
January 2025	16 (5.5)
February 2025	10 (3.4)
March 2025	10 (3.4)
April 2025	36 (12.4)
May 2025	40 (13.7)
June 2025	36 (12.4)
July 2025	40 (13.7)
August 2025	45 (15.5)
September 2025	21 (7.2)
October 2025	13 (4.5)

Note: Of the 286 unique authors, five authors contributed two posts each; no author contributed posts to both subreddits. “Unspecified GenAI tool” indicates that GenAI use was described, but no specific tool or platform was named. Percentages are based on 291 posts, except for author characteristics, which are based on 286 unique authors.

Table S5. Number of coded quotations, posts, and unique authors for each final code

Final code	Coded quotations, n			Posts, n	Unique authors, n
	r/Ask Docs	r/Chat GPT	Total		
Medical visit	56	77	133	125	125
Physical symptoms	76	33	109	109	109
Legitimacy	24	52	76	74	74
AI use for interpretation	31	31	62	59	59
Diagnostics	34	15	49	47	47
AI use for mental health	3	46	49	48	47
Outcome	6	42	48	44	44
AI-suggested diagnosis	23	24	47	47	47
AI use for symptom appraisal	30	17	47	46	46
Clinician diagnosis	18	22	40	40	39
AI use for recommendation	12	26	38	38	38
AI use for self-management	3	30	33	32	31
Healthcare access barrier	13	19	32	32	31
Medical dissatisfaction	11	15	26	26	26
AI use linked to healthcare seeking (explicitly stated)	6	18	24	23	23
Emotional response	20	3	23	23	23
Health behavior	4	17	21	21	21
Perceived AI benefit	0	19	19	19	19
Emotional support from AI	2	17	19	18	17
AI overreliance	0	18	18	17	17
Medical treatment	3	15	18	18	18
AI use for verification	12	6	18	18	18
Mental health symptoms	3	14	17	16	16
Personification of AI	2	15	17	16	16
AI use for tracking health data	3	12	15	15	15
AI use for medical visit preparation	2	13	15	15	13
Unresolved diagnosis	8	7	15	14	14
Comparing AIs	1	13	14	14	14
AI-physician disagreement	9	5	14	12	12
Self-diagnosis	10	2	12	12	12
Self-medication	9	1	10	10	9
AI use linked to treatment actions (explicitly stated)	4	6	10	10	10
AI-physician agreement	3	6	9	9	9
AI dissatisfaction	1	8	9	8	8
Psychological vulnerability	2	6	8	8	8
Limited social support	2	5	7	7	7
AI use for healthcare navigation	2	5	7	7	7
AI limitation	0	7	7	6	6
Confusion	5	2	7	7	7
No medical visit	2	4	6	6	6
Paying for AI	0	6	6	5	5
Doctor shopping	4	1	5	5	5
Patient-physician conflict	5	0	5	5	5

AI use while waiting for appointment	3	1	4	4	4
Comparing AI with Google	2	2	4	4	4
Privacy concern	0	4	4	4	4
Healthcare avoidance	1	3	4	2	2

Note: Because the same post or author may contribute to multiple codes, post and author counts are not mutually exclusive across codes and should not be summed.

File S1. SQL queries used to retrieve posts meeting the study criteria

AskDocs

```sql
SELECT DISTINCT id, title, body, created_at, author_id
FROM `rddt-eng-rfrexternal1-prod.for_researchers_external.posts`
WHERE subreddit_id = 't5_2xtuc'
AND created_at BETWEEN '2024-11-01' AND '2025-10-30'
AND (LOWER(title) LIKE '%health%' OR LOWER(body) LIKE '%health%'
OR LOWER(title) LIKE '%symptom%' OR LOWER(body) LIKE '%symptom%'
OR LOWER(title) LIKE '%doctor%' OR LOWER(body) LIKE '%doctor%'
OR LOWER(title) LIKE '%medical%' OR LOWER(body) LIKE '%medical%')
AND (LOWER(title) LIKE '%chatgpt%' OR LOWER(body) LIKE '%chatgpt%'
OR LOWER(title) LIKE '%gemini%' OR LOWER(body) LIKE '%gemini%'
OR LOWER(title) LIKE '%claude%' OR LOWER(body) LIKE '%claude%'
OR LOWER(title) LIKE '%generative ai%' OR LOWER(body) LIKE '%generative ai%'
OR LOWER(title) LIKE '%gpt%' OR LOWER(body) LIKE '%gpt%')
AND body NOT LIKE '%[deleted]%'
AND body NOT LIKE '%[removed]%'
AND author_id IS NOT NULL
ORDER BY created_at ASC
```

ChatGPT

```sql
SELECT DISTINCT id, title, body, created_at, author_id
FROM `rddt-eng-rfrexternal1-prod.for_researchers_external.posts`
WHERE subreddit_id = 't5_7hqomg'
AND created_at BETWEEN '2024-11-01' AND '2025-10-30'
AND (LOWER(title) LIKE '%health%' OR LOWER(body) LIKE '%health%'
OR LOWER(title) LIKE '%symptom%' OR LOWER(body) LIKE '%symptom%'
OR LOWER(title) LIKE '%doctor%' OR LOWER(body) LIKE '%doctor%'
OR LOWER(title) LIKE '%medical%' OR LOWER(body) LIKE '%medical%')
AND (LOWER(title) LIKE '%chatgpt%' OR LOWER(body) LIKE '%chatgpt%'
OR LOWER(title) LIKE '%gemini%' OR LOWER(body) LIKE '%gemini%'
OR LOWER(title) LIKE '%claude%' OR LOWER(body) LIKE '%claude%'
OR LOWER(title) LIKE '%generative ai%' OR LOWER(body) LIKE '%generative ai%'
OR LOWER(title) LIKE '%gpt%' OR LOWER(body) LIKE '%gpt%')
AND body NOT LIKE '%[deleted]%'
AND body NOT LIKE '%[removed]%'
AND author_id IS NOT NULL
ORDER BY created_at ASC
```
